# Longitudinal Associations Between Taste Sensitivity, Taste Liking, Dietary Intake and BMI in Adolescents

**DOI:** 10.3389/fpsyg.2021.597704

**Published:** 2021-02-18

**Authors:** Afroditi Papantoni, Grace E. Shearrer, Jennifer R. Sadler, Eric Stice, Kyle S. Burger

**Affiliations:** ^1^Department of Nutrition, Gillings School of Global Public Health, University of North Carolina at Chapel Hill, Chapel Hill, NC, United States; ^2^Biomedical Research Imaging Center, University of North Carolina at Chapel Hill, Chapel Hill, NC, United States; ^3^Department of Psychiatry and Behavioral Sciences, Stanford University, Stanford, CA, United States

**Keywords:** sugar, fat, hedonic ratings, dietary intake, adolescents

## Abstract

Taste sensitivity and liking drive food choices and ingestive behaviors from childhood to adulthood, yet their longitudinal association with dietary intake and BMI is largely understudied. Here, we examined the longitudinal relationship between sugar and fat sensitivity, sugar and fat liking, habitual dietary intake, and BMI percentiles in a sample of 105 healthy-weight adolescents (baseline: BMI %tile 57.0 ± 24.3; age 14–16 years) over a 4-year period. Taste sensitivity was assessed via a triangle fat and sweet taste discrimination test. Taste liking were rated on a visual analog scale for four milkshakes that varied in sugar and fat contents (high-fat/high-sugar (HF/HS), low-fat/high-sugar (LF/HS), high-fat/low-sugar (HF/LS), low-fat/low-sugar (LF/LS) milkshakes). A modified version of the reduced Block Food Frequency Questionnaire (BFFQ) was used to assess dietary intake. All measurements were repeated annually. Repeated measures correlations and linear mixed effects models were used to model the associations between the variables. Sugar sensitivity was negatively associated with liking for the LF/HS milkshake over the 4-year period. Low sugar sensitivity at baseline predicted increases in BMI percentile over time, but this association didn’t survive a correction for multiple comparisons. Percent daily intake from fat was positively associated with liking for the HF/HS milkshake and negatively associated with liking for the LF/LS milkshake over 4 years. Together, these results demonstrate that lower sensitivity to sweet taste is linked to increased hedonic response to high-sugar foods and increased energy intake from fat seems to condition adolescents to show increased liking for high-fat/high-sugar foods.

## Introduction

Obesity is a complex disease state largely driven by overconsumption of energy-dense foods resulting in a positive energy balance (Mitchell et al., 2011). Taste perception and sensitivity influence consumption since they identify and evaluate foods suitable for ingestion (Loper et al., 2015). These sensory aspects of food intake shape both acute food choices and long-term ingestive behavior from childhood through adulthood providing the foundation for weight regulation (Drewnowski, 1997; Mennella et al., 2005). As such, understanding the impact of taste perception of foods on food liking and intake is a critical point for determining the development of and thus prevention of impaired body weight maintenance and obesity.

Taste sensitivity can be described as the minimum concentration at which a person is able to perceive a specific taste quality (Sørensen et al., 2003). Taste liking, on the other hand, is a measure of the affective component of attitude and is linked to the hedonic value of each taste quality (Drewnowski et al., 2012). To date, evidence linking taste sensitivity to taste liking is either limited or inconclusive (Tan and Tucker, 2019). Sensitivity to sweet taste is the construct studied most in children and adults (Hardikar et al., 2017). Studies in children report a fragile, inverse relation between sweet taste sensitivity and sweet taste liking (Fry Vennerød et al., 2018). In contrast, studies in adolescents and adults have failed to find a consistent relationship between sweet taste sensitivity and liking (Mojet et al., 2005; Coldwell et al., 2009; Garneau et al., 2018). The lack of an observed relationship among older participants could be attributed to the well-established reduction in sweet taste liking observed from childhood to adolescence and adulthood (Mennella et al., 2011; Mennella and Bobowski, 2015). Yet data suggest that sweet taste sensitivity appears to increase with age (Joseph et al., 2016). The changes observed in sweet taste sensitivity and sweet taste liking over the course of development, which support the hypothesis that taste sensitivity and preferences are dynamic, may be a function of growth. Another viable hypothesis could be that these trends are a function of repeated exposures to sweet foods via frequent intake, with the ability to discern the intensity of sweet taste increasing over time, but the hedonic pleasantness derived from sweet foods decreasing (Epstein et al., 2009). The relationship between fat taste sensitivity and fat liking has not been studied extensively, with one study reporting a negative association among adults (Bolhuis et al., 2016) and another failing to detect an association (Chamoun et al., 2019). Given the inconsistent findings across studies, longitudinal study designs across different life stages are warranted.

Taste sensitivity and its association with dietary intake have been studied to a lesser extent. Sweet taste sensitivity has been linked to food intake, with highly sweet-sensitive adults consuming more dietary protein and less carbohydrates (Han et al., 2017), and reporting lower energy intake per 7-day food diary (Martinez-Cordero et al., 2015). However, other studies found no significant link between sweet taste sensitivity and dietary intake among adults (Low et al., 2016; Jayasinghe et al., 2017). In addition, several studies reported a clear association between lower fat sensitivity and greater high-fat food intake (Costanzo et al., 2017; Heinze et al., 2018), while intake of foods rich in fiber and vitamins is lower (Heinze et al., 2018). Few studies have examined the association between fat sensitivity and food intake in adolescents. With unclear support for associations between taste sensitivity and dietary intake, further research is needed to determine the relation.

The relationship between taste liking and dietary intake has been more widely studied. Dietary intake is determined by multiple factors including biological (hunger, taste preference), psychological (perceived stress, anxiety, mood), and socioeconomic (familial environment, food availability, income, education, culture) components (Leng et al., 2017). However, taste liking and food palatability seem to be some of the key drivers of food choice (Chen and Antonelli, 2020). This is particularly true for adolescents, where hunger, food cravings and taste liking are consistently the most important determinants of food choices (Neumark-Sztainer et al., 1999; Krølner et al., 2011). Hedonic measurements of sweet taste liking have been associated with greater total energy intake (Costanzo et al., 2017), carbohydrate intake (Smith et al., 2016), and both refined and total sugar intake (Holt et al., 2000). Additionally, people classified as sweet “likers” consume more calories from sugar-sweetened beverages (Garneau et al., 2018) and have lower fiber intake (Turner-McGrievy et al., 2013). Although, this relationship has not been observed in all studies (Sartor et al., 2011; Stevenson et al., 2016). The mixed findings could be due to the smaller sample size compared to the studies with positive associations or to differences in study design. Studies have also found a positive relationship between fat liking and high-fat food intake (Ricketts, 1997; Park H. et al., 2018), with fat liking being linked to greater consumption of saturated fats and desserts, and lower consumption of fruits, vegetables, and omega-3 fatty acids (Méjean et al., 2014). Additionally, in a prospective study, higher fat liking was a risk factor for future obesity onset, with the relationship predominantly explained by greater overall dietary intake (Lampuré et al., 2016).

Taste sensitivity and taste liking have also been associated with weight status, albeit predominantly cross-sectionally (Noel et al., 2017; Tucker et al., 2017; Vignini et al., 2019; Sobek et al., 2020; Venditti et al., 2020). Higher BMI is related to lower sensitivity to sweet taste (Noel et al., 2017; Vignini et al., 2019), while a recent meta-analysis showed that fat sensitivity was not related to weight status in adults (Tucker et al., 2017). In turn, there is weak evidence that liking for fat or sweet taste separately is associated with higher BMI (Sobek et al., 2020; Venditti et al., 2020), while higher liking for fat-and-sweet sensations has been associated with an increased risk of obesity in women (Salbe et al., 2004; Lampuré et al., 2016).

Together, these studies provide some evidence about the associations between taste sensitivity, taste liking, dietary intake and BMI, but the lack of longitudinal designs limits the ability to draw inferences about the nature of these associations. Hence, the present analysis aimed to assess the relationships between sweet taste and fat sensitivity, sweet taste and fat liking, food intake and BMI percentile in a sample of lean adolescents (14–16 years old) at baseline and over a 3-year follow-up period. We hypothesized that sweet taste and fat sensitivity would be negatively associated with sweet taste and fat liking over the 4-year study period. Additionally, we expected that lower sensitivity and higher liking for sweet taste and fat would be associated with greater food intake at both baseline and during follow-up. Further we hypothesized that lower sweet taste sensitivity and higher sweet taste liking at baseline would predict increases in BMI percentiles over the 4-year study period.

## Materials and Methods

### Participants

Participants were recruited as part of a longitudinal randomized controlled study investigating neurobehavioral responses to palatable food images and receipt of chocolate milkshakes at baseline and three annual follow-up visits (Stice et al., 2013; Sadler et al., 2019). Participants were eligible for the study if they were between 14 and 16 years old and had a BMI-for-age percentile between 25th and 75th at baseline. Further details about the sample, recruitment, and complete study procedures are detailed elsewhere (Stice et al., 2013; Sadler et al., 2019). Exclusion criteria included reports of binge eating or compensatory behavior in the past 3 months, use of psychotropic medications or illicit drugs, head injury with loss of consciousness, or an axis I psychiatric disorder diagnosis in the past year (including anorexia nervosa, bulimia nervosa, or binge eating disorder), and dairy allergies. At each annual assessment, data collection for all eligible participants was completed over two separate study visits, on average 17 ± 16 days apart, with anthropometrics, taste sensitivity, and dietary intake being measured during the first visit and taste liking measured during the second visit. Parents provided written informed consent and adolescents provided written assent. This study was approved by the Oregon Research Institute’s Institutional Review Board and is registered at www.clinicaltrials.gov as NCT01949636.

### Anthropometrics and Demographics

Height was measured to the nearest mm using a stadiometer. Weight was assessed to the nearest 0.1 kg using a digital scale with participants wearing light clothing, without shoes. BMI values (kg/m^2^) were calculated at baseline and at 1-, 2-, and 3-year follow ups. BMI percentiles were derived for participants based on the Center for Disease Control (CDC) growth charts (Kuczmarski et al., 2002).

### Internal State Ratings

To standardize hunger and fullness levels, participants rated their hunger and fullness on a VAS scale from 0 (*“I am not hungry/full at all”*) to 20 (*“I have never been more hungry/full”*) prior to the taste sensitivity and the taste liking assessments. In the case of the taste liking test, if a hunger score ≥ 17 was indicated, subjects were offered a snack to bring their hunger to a neutral state (20% of subjects received a snack at year 1, 17.1% at year 2, 10.5% at year 3, and 20% at year 4). A second VAS was performed to confirm the snack was effective in normalizing their hunger/fullness.

### Taste Sensitivity

At each annual assessment, taste sensitivity was assessed during the behavioral visit. Triangle taste discrimination tests (Pepino et al., 2010) assessed fat and sweet taste sensitivity respectively. For the fat sensitivity test, participants had to discriminate between six possible solutions (solutions A–F) of chocolate milk with varying fat content. For the sweet taste sensitivity test, participants had to discriminate between six possible solutions (solutions A–F) of Kool-Aid with varying sugar content. The formulation of the solutions A–F used in the triangle taste discrimination tests can be found in Supplementary Table 2. The administration order of the fat and sweet taste sensitivity tests was counterbalanced across participants. For each test, participants were presented with three 8 fl oz cups, two containing stimuli with identical sugar or fat concentrations and one containing a different sugar or fat concentration. For the first trial, they tasted all three and chose the one that was different. If they chose correctly two times, they moved on to a more difficult trial where the difference in concentration between the two identical and the one different stimulus was smaller. If they chose incorrectly two times, that trial was terminated and they moved on to an easier trial. This process was repeated until there were no trials left (maximum of five trials) or they failed to identify the different stimulus twice in the easiest trial. The number of times that participants correctly discriminated between the stimuli served as their taste sensitivity score for each test, with a possible range of 0 (least sensitive) to 5 (most sensitive). Detailed instructions for the triangle test can be found in Supplementary Figure 3. Each participant rinsed with water between each sample.

### Taste Liking

Taste liking was assessed during the second visit at each annual assessment. Participants were asked to refrain from eating or drinking (except water) for at least 4 h prior to their scheduled visit. Participants rated the pleasantness (“*How pleasant is this taste?*”) of four milkshakes that varied in sugar and fat contents. Detailed description of the milkshake contents can be found elsewhere (Stice et al., 2013), but in brief, each milkshake included the same ice-cream base and chocolate syrup. Fat contents of the milkshakes varied by the type of milk (half and half compared with 2% milk). The sweetness varied by the simple-syrup content. We investigated the taste liking for the following milkshakes: a high-fat/high-sugar (HF/HS) milkshake (170 kcal, 7.5g fat, and 23 g sugar/100 mL), a low-fat/high-sugar (LF/HS) milkshake (124 kcal, 1.9 g fat, 23.7 g sugar/100 mL), a high-fat/low-sugar (HF/LS) milkshake (129 kcal, 9.0g fat, and 7.3g sugar/100 mL), and a low-fat/low-sugar (LF/LS) milkshake (74 kcal, 2.4g fat, and 8.7 g sugar/100 mL). The LF/HS and HF/LS milkshakes were designed such that they had similar energy densities (1.24 kcal/mL for the LF/HS milkshake compared with 1.29 kcal/mL for the HF/LS milkshake). For the ratings, participants sampled a small amount of each milkshake (order counterbalanced) and rated the pleasantness on a visual analog scale (VAS) that ranged from 0 (“*most unpleasant sensation ever”*) to 20 (“*most pleasant sensation ever”*).

### Dietary Intake

A modified version of the reduced Block Food Frequency Questionnaire (BFFQ) (Block et al., 1990) was used to assess average dietary intake. Across all food items, participants were given a definition of a medium portion of that food item and asked to indicate the frequency of consumption over the previous 2-week period. Response options ranged from 1 = “Never in the last 2 weeks” to 6 = “Daily or more in the last 2 weeks.” Daily caloric intake, percent daily caloric intake from fat, and percent daily caloric intake from sugar were estimated from BFFQ responses.

### Statistical Analysis

Statistical analyses were performed using R (version 3.6.1, The R Foundation for Statistical Computing). Descriptive statistics to summarize means, standard deviation, and percentages were generated for variables of interest. Repeated measures correlations were used to examine the within-individual longitudinal relationship between fat and sweet taste sensitivity, fat and sweet taste liking, daily caloric intake, percent daily caloric intake from fat, and percent daily caloric intake from sugar (package *rmcorr* version 0.3.0). To assess the change in BMI percentiles over time, a linear line was fit to measurements of BMI percentile at years 1, 2, 3, 4 for each participant. The slope of the line was considered the change in BMI percentile over the 4 years. BMI percentile change was also modeled using a quadratic term, but the resulting model did not significantly improve fit, as assessed by the Akaike’s Information Criteria, so the linear slope was used in analyses. Linear regression was used to test whether 4-year change in BMI percentile (slope) was predicted by baseline sensitivity and liking for fat and sweet taste, controlling for sex, baseline BMI percentile, age and hunger. Results were corrected for multiple comparisons using the Benjamini–Hochberg procedure (pFDR < 0.05).

### *Post hoc* Analysis

Significant and marginally significant results from the correlations were further explored using linear mixed effects models with maximum likelihood estimation (package *nlme* version 3.1-140). The baseline models included the outcomes of interest (pleasantness for HF/HS, LF/HS, and LF/LS milkshakes) and the predictors as fixed effects (sweet taste sensitivity, percent daily caloric intake from fat). To account for individual differences in the outcomes, random intercepts were included in the model at the subject level. Additional confounding variables were added as fixed effects in a stepwise manner: time, sex, BMI, age, daily caloric intake (only for the models with the percent daily caloric intake from fat), hunger, fullness. However, the addition of age, daily caloric intake, hunger, and fullness neither improved model fit nor changed the significant results, hence, the linear mixed model results presented below include only time, sex, and BMI as covariates. Statistical significance was set at *p* < 0.05.

## Results

### Participants Characteristics

Participant demographics and summary of behavioral variables can be found in Table 1. Of the 125 participants that had complete anthropometric and behavioral data at baseline, 105 participants had complete taste liking and taste sensitivity data over the 4-year study period, of which 85 had complete dietary intake data. Demographics, anthropometrics and behavioral variables did not differ between the total sample (*n* = 105) and the subsample of 85 participants used for the dietary intake analysis, with the exception of hunger at year 3 and fullness at year 4, both being higher in the subsample. Complete demographics for the subsample (*n* = 85) can be found in Supplementary Table 1. The total sample (*n* = 105) consisted of 47 (44.8%) male and 58 (55.2%) female adolescents [age = 15 ± 1 (14 – 16) years at baseline]. All adolescents were healthy-weight at baseline [25th – 75th BMI-for-age percentile; BMI = 21.2 ± 2.3 (16.2 – 26.4)].

**TABLE 1:** Participant (*n* = 105) characteristics and behavioral measures.

	**Year 1 Visit (Baseline)**	**Year 2 Visit**	**Year 3 Visit**	**Year 4 Visit**

	**Count (Percent)**			
**Sex**				
*Male*	47 (44.8)			
*Female*	58 (55.2)			
**Race**				
*Asian*	5 (4.8)			
*Black or African American*	7 (6.7)			
*White*	83 (79.0)			
*More than one race*	5 (4.8)			
*Other or Missing*	5 (4.8)			
	*Mean* ± *SD (Range)*			
**Age (years)**	15 ± 1 (14–16)			
**BMI (kg/m^2^)**	21.2 ± 2.3 (16.2–26.4)	21.5 ± 2.6 (16.8–28.3)	22.0 ± 2.8 (17.0–31.3)	22.7 ± 3.5 (16.2–40.6)
BMI percentile*	57.0 ± 24.3 (5.4–94.9)	53.8 ± 25.6 (2.4–94.7)	52.4 ± 26.5 (1.7–97.3)	52.6 ± 27.5 (0.5–99.7)
**Taste Sensitivity**				
*Fat*	2.37 ± 1.19 (0–5)	2.30 ± 1.15 (0–5)	2.43 ± 1.07 (0–5)	2.63 ± 1.25 (0–5)
*Sweet*	2.81 ± 1.03 (0–5)	2.77 ± 0.93 (0–5)	2.87 ± 1.08 (0–5)	2.88 ± 1.03 (0–5)
**Taste Liking** (pleasantness rating)				
*HF/HS*	14.61 ± 3.18 (7–20)	13.22 ± 4.31 (2–20)	13.74 ± 3.87 (1–20)	12.97 ± 4.53 (0–20)
*LF/HS*	11.94 ± 3.91 (1.5–20)	11.89 ± 4.04 (1.5–19.5)	13.09 ± 3.69 (0.5–19)	12.75 ± 3.68 (1–19.5)
*HF/LS*	12.87 ± 4.31 (1.5–20)	12.94 ± 4.34 (0.5–20)	12.24 ± 4.61 (1–19.5)	12.35 ± 4.07 (1–20)
*LF/LS*	10.10 ± 3.87 (0–19.5)	11.57 ± 3.79 (0–17.5)	11.35 ± 3.35 (2–18.5)	11.49 ± 3.67 (1–19)
Dietary Intake**				
*Daily caloric intake (kcal)*	1861 ± 313 (1303–3159)	1888 ± 331 (1388–3173)	1827 ± 285 (1297–2903)	1837 ± 340 (1211–2901)
*% daily caloric intake from fat*	35.4 ± 1.4 (32–38)	35.4 ± 1.6 (31–40)	35.2 ± 1.5 (31–38)	35.2 ± 1.5 (31–38)
*% daily caloric intake from sugar*	13.6 ± 1.4 (10–18)	13.6 ± 1.4 (11–18)	13.6 ± 1.6 (11–19)	13.2 ± 1.4 (11–19)
**Hunger**				
Prior to Taste Sensitivity test	8.82 ± 4.99 (0–20)	9.86 ± 4.55 (0–17.5)	9.90 ± 4.34 (0–18)	11.03 ± 3.82 (0–17.5)
Prior to Taste Liking test	11.21 ± 3.95 (0–19.5)	11.31 ± 4.26 (0–19)	11.46 ± 4.10 (0–20)	12.07 ± 3.60 (1–18.5)
**Fullness**				
Prior to Taste Sensitivity test	9.06 ± 4.35 (0–19)	8.06 ± 4.05 (0–18.5)	7.88 ± 4.28 (0–18)	7.05 ± 3.90 (0–17.5)
Prior to Taste Liking test	6.80 ± 4.39 (0–20)	6.60 ± 4.40 (0–18.5)	6.20 ± 3.97 (0–15.5)	5.99 ± 3.94 (0–17)

*^∗^*n* = 101; **^∗∗^***n* = 85.*

*HF/HS, high-fat/high-sugar milkshake; LF/HS, low-fat/high-sugar milkshake; HF/LS, high-fat/low-sugar milkshake; LF/LS, low-fat/low-sugar milkshake.*

### Taste Sensitivity and Taste Liking Associations

**FIGURE 1:**
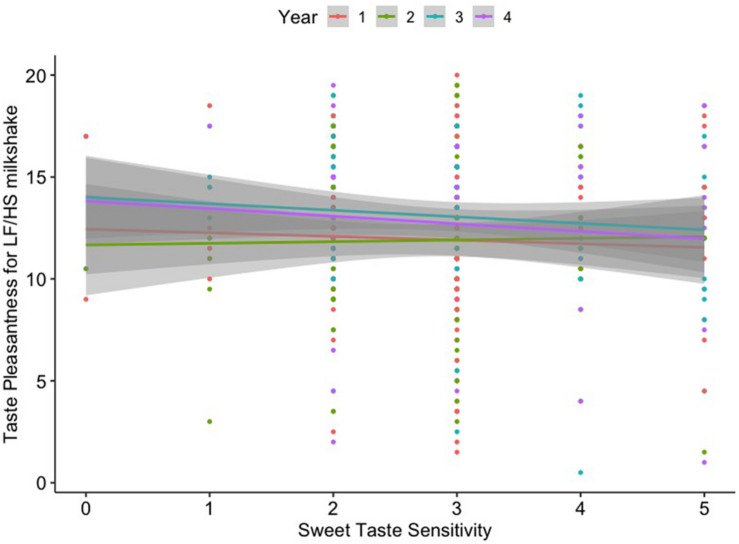
Linear relationship between sweet taste sensitivity and taste liking (pleasantness) for the low-fat/high-sugar (LF/HS) milkshake across 4 years.

Sweet taste sensitivity and fat sensitivity were stable over time (*p* > 0.05 for the effect of time across all 4 years). Sweet taste sensitivity negatively correlated with pleasantness (*r* = –0.188, *p* < 0.001, pFDR = 0.021) for the LF/HS milkshake over the 4-year study period. Fat sensitivity did not correlate with pleasantness ratings for any of the four milkshakes over the 4-year period. Additionally, sweet taste sensitivity was not significantly associated with fat sensitivity over time (*r* = 0.029, *p* = 0.613). Repeated measures correlation results are displayed in Table 2 and Supplementary Figure 1. The effect of sweet taste sensitivity on pleasantness for the LF/HS milkshake over time remained significant after controlling for confounding variables in the linear mixed model [β = –0.46, 95% CI = (–0.76, –0.16), *p* = 0.003] (Table 3 and Figure 1).

**TABLE 2:** Repeated measures correlations between taste sensitivity and taste liking.

		**Taste Sensitivity**			
		***Sweet Taste***		***Fat***	
		***r***	***p***	***r***	***p***
**Taste Liking** (pleasantness rating)	*HF/HS*	0.004	0.944	0.021	0.709
	*LF/HS*	–0.188*	**<0.001**	0.035	0.534
	*HF/LS*	–0.064	0.254	–0.006	0.911
	*LF/LS*	–0.087	0.122	–0.024	0.672

*^∗^pFDR < 0.05.*

*Degrees of freedom: 314.*

**TABLE 3:** Results of the linear mixed models for taste liking (pleasantness) with sweet taste sensitivity.

***Outcomes***	**Pleasantness for LF/HS milkshake**		
***Predictors***	**β *estimates***	***95% CI***	***p***
(Intercept)	18.01	14.64 – 21.37	**<0.001**
Sweet Taste Sensitivity	–0.46	–0.76 – 0.16	**0.003**
Year 1	*REF*		
Year 2	0.00	–0.75 – 0.75	0.998
Year 3	1.34	0.59 – 2.10	**<0.001**
Year 4	1.13	0.35 – 1.91	**0.005**
Male	*REF*		
Female	–1.38	–2.51 – 0.25	**0.019**
BMI	–0.19	–0.34 – 0.04	**0.015**
**Random Effects**			
*SD*	2.56		
CI_sd_	2.14 – 3.06		
N_grp_	105		

*Bold values represent findings with an uncorrected *p*-value < 0.05.*

### Dietary Intake and Associations With Taste Sensitivity and Liking

**FIGURE 2:**
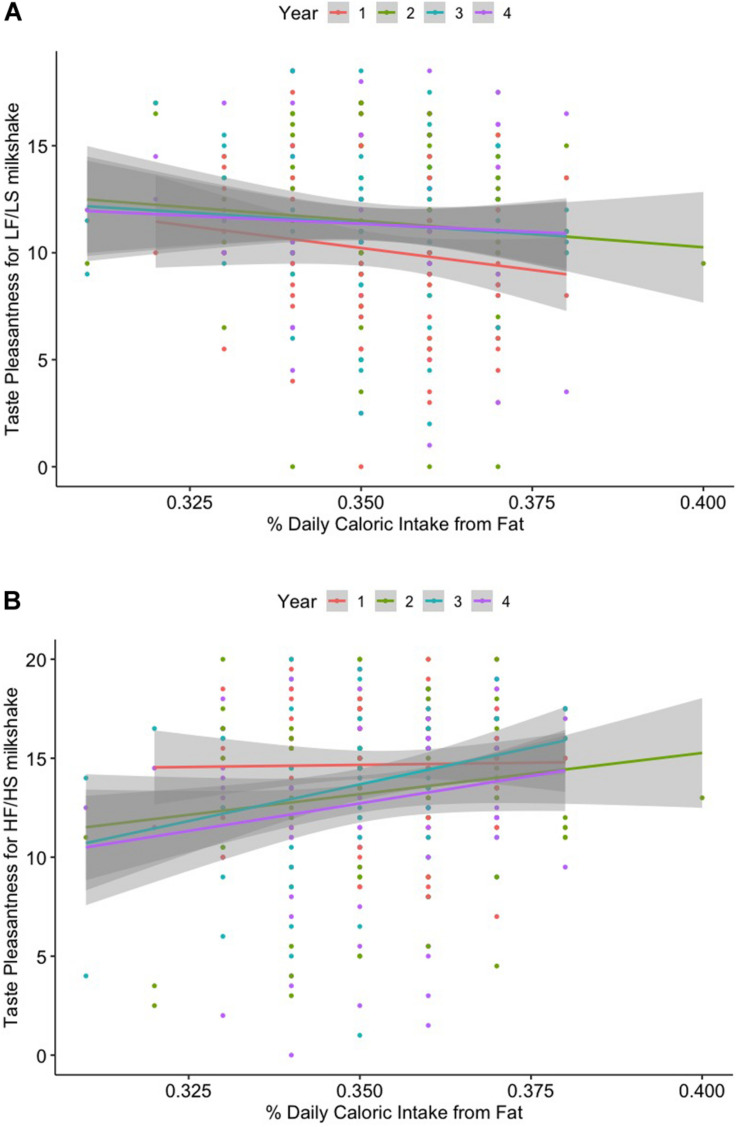
**(A)** Linear relationship between percent daily caloric intake from fat and taste liking (pleasantness) for the high-fat/high-sugar (HF/HS) milkshake. **(B)** Linear relationship between percent daily caloric intake from fat and taste liking (pleasantness) for the low-fat/low-sugar (LF/LS) milkshake across 4 years.

Percent daily caloric intake from fat was positively correlated with pleasantness for the HF/HS milkshake (*r* = 0.132, *p* = 0.035) over the 4-year study period. Although there was only weak evidence of a relationship (*p* = 0.051), percent daily caloric intake from fat had a small negative correlation with pleasantness for the LF/LS milkshake (*r* = –0.122) over the 4 years. However, these results failed to survive correction for multiple comparisons (pFDR = 0.411 for both). Neither sweet taste nor fat sensitivity was associated with dietary intake over time. Results are displayed in Table 4 and Supplementary Figures 2a,b. The effect of % daily caloric fat intake on future pleasantness for the HF/HS milkshake over time remained significant after controlling for confounding variables in the linear mixed model [β = 29.53, 95% CI = (5.75, 53.31), *p* = 0.016]. Lastly, the effect of % daily caloric fat intake on future pleasantness for the LF/LS milkshake over time was also significant [β = −27.13, 95% CI = (−52.43, −1.82), *p* = 0.038] (Table 5 and Figures 2A,B).

**TABLE 4:** Repeated measures correlations between dietary intake and taste sensitivity and liking.

		**Dietary Intake**					
		***Daily Caloric Intake***		***% Daily Caloric Intake from Fat***		***% Daily Caloric Intake from Sugar***	
		***r***	***p***	***r***	***p***	***r***	***p***
**Taste Liking** (pleasantness rating)	*HF/HS*	0.003	0.959	**0.132**	**0.035**	0.011	0.867
	*LF/HS*	–0.117	0.061	0.032	0.608	–0.065	0.302
	*HF/LS*	0.063	0.315	0.090	0.152	–0.021	0.734
	*LF/LS*	–0.076	0.225	–0.122	0.051	0.005	0.937
**Taste Sensitivity**	*Sweet Taste*	0.105	0.094	0.016	0.800	–0.015	0.806
	*Fat*	0.056	0.371	–0.061	0.329	0.014	0.822

*Results did not survive FDR correction. Degrees of freedom: 254. Bold values represent findings with an uncorrected p-value < 0.05.*

**TABLE 5:** Results of the linear mixed models for taste liking (pleasantness) with % daily caloric intake from fat.

***Outcomes***	**Pleasantness for HF/HS milkshake**			**Pleasantness for LF/LS milkshake**		
***Predictors***	**β *estimates***	***95% CI***	***p***	**β *estimates***	***95% CI***	***p***
(Intercept)	4.20	–4.70 – 13.10	0.358	19.69	10.33 – 29.06	**<0.001**
% daily caloric intake from fat	29.53	5.75 – 53.31	**0.016**	–27.13	–52.43 – 1.82	**0.038**
Year 1	*REF*			*REF*		
Year 2	–1.35	–2.20 – 0.49	**0.002**	1.33	0.39 – 2.28	**0.006**
Year 3	–0.83	–1.70 – 0.03	0.061	1.21	0.26 – 2.16	**0.014**
Year 4	–1.86	–2.75 – 0.97	**<0.001**	1.18	0.21 – 2.15	**0.018**
Male	*REF*			*REF*		
Female	–1.90	–3.15 – 0.66	**0.004**	–0.91	–1.96 – 0.13	0.089
BMI	0.05	–0.12– 0.22	0.558	0.02	–0.14 – 0.18	0.789
**Random Effects**						
*SD*	2.49			1.82		
CI_sd_	2.04 – 3.05			1.39 – 2.38		
N_grp_	85			85		

*Bold values represent findings with an uncorrected *p*-value < 0.05.*

### Prediction of BMI Percentile Change by Taste Sensitivity and Liking

**FIGURE 3:**
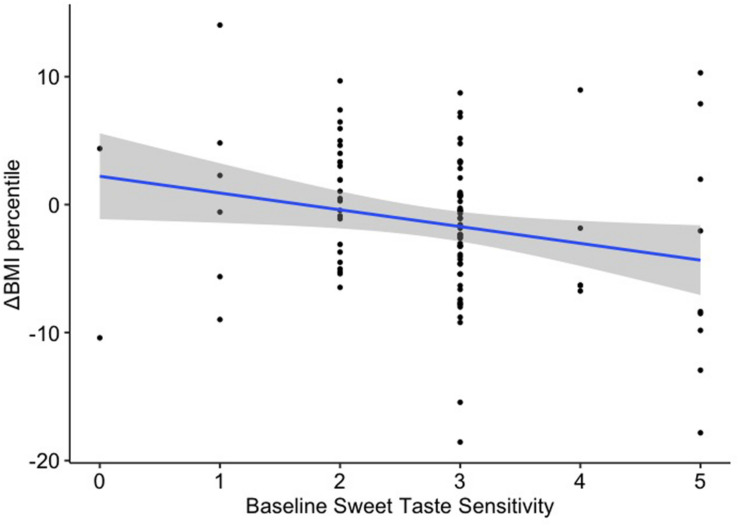
Linear regression between baseline sweet taste sensitivity and change in BMI percentile over 4 years.

Sweet taste sensitivity at baseline was a significant predictor of BMI percentile change [β = –1.28, 95% CI = (–2.41, –0.15), *p* = 0.026; Figure 3], although it failed to survive corrections for multiple comparisons (pFDR = 0.157). Fat sensitivity, sweet taste and fat liking were not significantly associated with changes in BMI percentile over the 4-year study period (Table 6).

**TABLE 6:** Results of the linear regression models of taste liking and taste sensitivity with change in BMI percentile over 4 years.

	**ΔBMI percentile**		
***Predictors***	**β *estimates***	***95% CI***	***p***
**Taste Sensitivity**			
Fat	–0.21	–1.21 – 0.80	0.682
Sweet	–1.28	–2.41 – 0.15	**0.026**
**Taste Liking** (pleasantness rating)			
HF/HS	0.06	–0.32 – 0.45	0.750
LF/HS	0.03	–0.28 – 0.34	0.850
HF/LS	–0.01	–0.29 – 0.28	0.960
LF/LS	–0.12	–0.42 – 0.19	0.438

*All models were controlled for sex, baseline BMI percentile, age, and hunger. Bold values represent findings with an uncorrected *p*-value < 0.05.*

## Discussion

Taste sensitivity and liking are important drivers of dietary choices particularly among adolescents who are experiencing an increase in food choice autonomy (Bassett et al., 2008). However, no study has assessed how these aspects of tastes change over time and their relation to dietary intake. Here, we observed that higher sweet taste sensitivity was associated with lower liking of a high-sugar/low-fat drink. These findings are consistent with previous studies in young adults (Chamoun et al., 2019). The negative association between sensitivity and hedonic evaluation of sweet taste supports the idea that these measures provide distinct but complementary information about taste sensations and food choices (Webb et al., 2015). Sugar has been repeatedly associated with promoting hedonically motivated eating behavior (e.g., compulsive eating), therefore, people with a high threshold for sweet taste discrimination may be insensitive to high sugar content in foods. This may place them at an increased risk for excessive sugar intake and impaired control over dietary intake (Kampov-Polevoy et al., 2006). In concert, high-sugar milkshake intake in the same group of adolescents elicited greater brain response in regions associated with food reward (e.g., putamen), oral somatosensation (e.g., Rolandic operculum), and gustatory stimulation (e.g., insula, thalamus) (Stice et al., 2013), suggesting that adolescents with lower sensitivity to high-sugar drinks have a greater reward physiological response.

The association between sweet taste sensitivity and liking did not extend to the high-sugar/high-fat milkshake. Given that texture and mouthfeel seem to also influence hedonic responses to fats (Drewnowski and Almiron-Roig, 2010), the addition of fat and its viscosity/mouth feel may impact the hedonic response to sugar, dissociating it from sweet taste sensitivity. This result is specific to the high-sugar/low-fat milkshake, so adolescents with lower sensitivity to high-sugar drinks may prefer high sugar beverages with lower-fat content, where the hedonic response to sugar is not obscured by fat. However, differences in sweet sensitivity were not associated with decreased fat intake, suggesting that other factors could have a greater influence on food choices.

Dietary intake from fats was positively associated with liking for a high-sugar/high-fat drink and negatively associated with liking for a low-sugar/low-fat drink. This dovetails multiple research studies in both children and adults (Ricketts, 1997; Park H. et al., 2018), whereas increased liking for fatty foods has been associated with high fat intake as well as low fiber and vegetable intake (Drewnowski and Hann, 1999). Adolescents who prefer fat may be less likely to consume healthier foods, such as fruits and vegetables, as they find them less tasty, and instead consume foods high in fat, leading to a positive energy balance. Frequently overlooked, dislike of energy-dense foods may be protective against weight gain (Sadler et al., 2019), potentially promoting a more ‘balanced’ diet. Food choices are critical during adolescence, when teenagers transition from a controlled food environment toward independent food-based decision making (Bassett et al., 2008). Thus, adolescents with increased fat intake at home are more likely to be conditioned to find high-fat/high-sugar foods more pleasant and consume more of these foods later in life, possibly contributing to excess weight gain.

Several studies have shown that lower sensitivity to fatty foods is linked to higher intake of high-fat foods (Stewart et al., 2011; Liang et al., 2012), contributing to excess fat intake in the long-term. However, we did not observe this finding in our sample. This difference may be due to the methodology used in the current study, such as unique sample characteristics or the variability in the fat content of the samples used in the taste sensitivity test. Furthermore, we did not observe an association between fat taste sensitivity and fat liking, which is in line with previous observations (Chamoun et al., 2019).

Although not significant after correction for multiple comparisons, it is noteworthy that baseline sweet taste sensitivity predicted BMI percentile change of the 4-year study period. Participants with lower sensitivity had a greater increase in BMI percentile. Adolescents with a dulled sensitivity to the sweet taste could be at an increased risk of long-term weight gain, as reductions in sweet taste sensitivity may contribute to an impaired satiety response, resulting in excess high-calorie food consumption, akin to many brain based models of weight gain (Volkow et al., 2008; Yokum and Stice, 2019). Surprisingly, whereas the sweet taste sensitivity and liking for high-sugar drinks were negatively associated, BMI percentile change was not predicted by baseline liking ratings, suggesting that taste sensitivity affects future weight through a mechanism independent of food liking.

It is important to consider the limitations of this study. The effect sizes for the significant repeated measures correlations were relatively small per Cohen (2013). Indeed, in larger sample studies, smaller, yet statistically significant effects can be observed. This indicates that, while the effect is present on a larger sample as a significant trend, it may be less meaningful on an individual level. Nevertheless, the findings from this study do provide novel information in the field of taste and weight regulation that can be used to inform future studies. Few levels of sugar and fat were tested, which may have provided a less precise test of taste sensitivity. Moreover, while the stimuli were designed to mimic ‘real-world’ foods, sensitivity may vary with different sweeteners (e.g., fructose) and types of fat (e.g., varied fatty acids). In addition, the fat sensitivity test used in this study included solutions of milk with varying fat content instead of solutions prepared with a single type of fatty acid, thus it did not allow us to differentiate whether participants made decisions based on basic taste (fatty acid) or other textural properties of these solutions. The validity of self-reported dietary intake is continually being debated, as it is susceptible to many biases (Park Y. et al., 2018). Also, the present study did not use one of the measures that are considered more valid (e.g., 7-day diet diary); as such, the diet data results need replication. Additionally, the current sample is quite homogeneous, while recent studies suggest there could be differences in taste sensitivity among racial and ethnic groups (Williams et al., 2016). Further research is needed to replicate these findings in more diverse samples. Despite these weaknesses, the large sample and the prospective collection of behavioral measures are meaningful strengths.

## Conclusion

In sum, these results point toward the notion that lower sensitivity to sweet taste is linked to increased hedonic response to high-sugar foods, with potential contributions to overeating. Further, increased energy from fat may act to ‘condition’ adolescents to show increased liking for high-fat/high-sugar foods. These data are supported by many brain-based models of obesity and provide a nuanced examination of sensitivity and liking. The consistency of the findings with previous literature point to the importance of aspects of taste intensity underlying food intake and possibly weight regulation.

## Data Availability Statement

The raw data supporting the conclusions of this article will be made available by the authors, without undue reservation.

## Ethics Statement

The studies involving human participants were reviewed and approved by Oregon Research Institute’s Institutional Review Board. Written informed consent to participate in this study was provided by the participants’ legal guardian/next of kin.

## Author Contributions

AP was responsible for data analysis and drafting the manuscript. GS contributed to data analysis. ES and KB were responsible for study design, data collection, and data curation. All the authors were responsible for reviewing and editing the manuscript, and read and approved the final manuscript.

## Conflict of Interest

The authors declare that the research was conducted in the absence of any commercial or financial relationships that could be construed as a potential conflict of interest.
