# Supplementary material for: Longitudinal Associations Between Taste Sensitivity, Taste Liking, Dietary Intake and BMI in Adolescents
Source: Front Psychol. 2021 Feb 18;12:597704. doi: 10.3389/fpsyg.2021.597704 (PMC7935517; doi:10.3389/fpsyg.2021.597704)
Supplement: Supplementary file 2 [file Image_2.PDF]

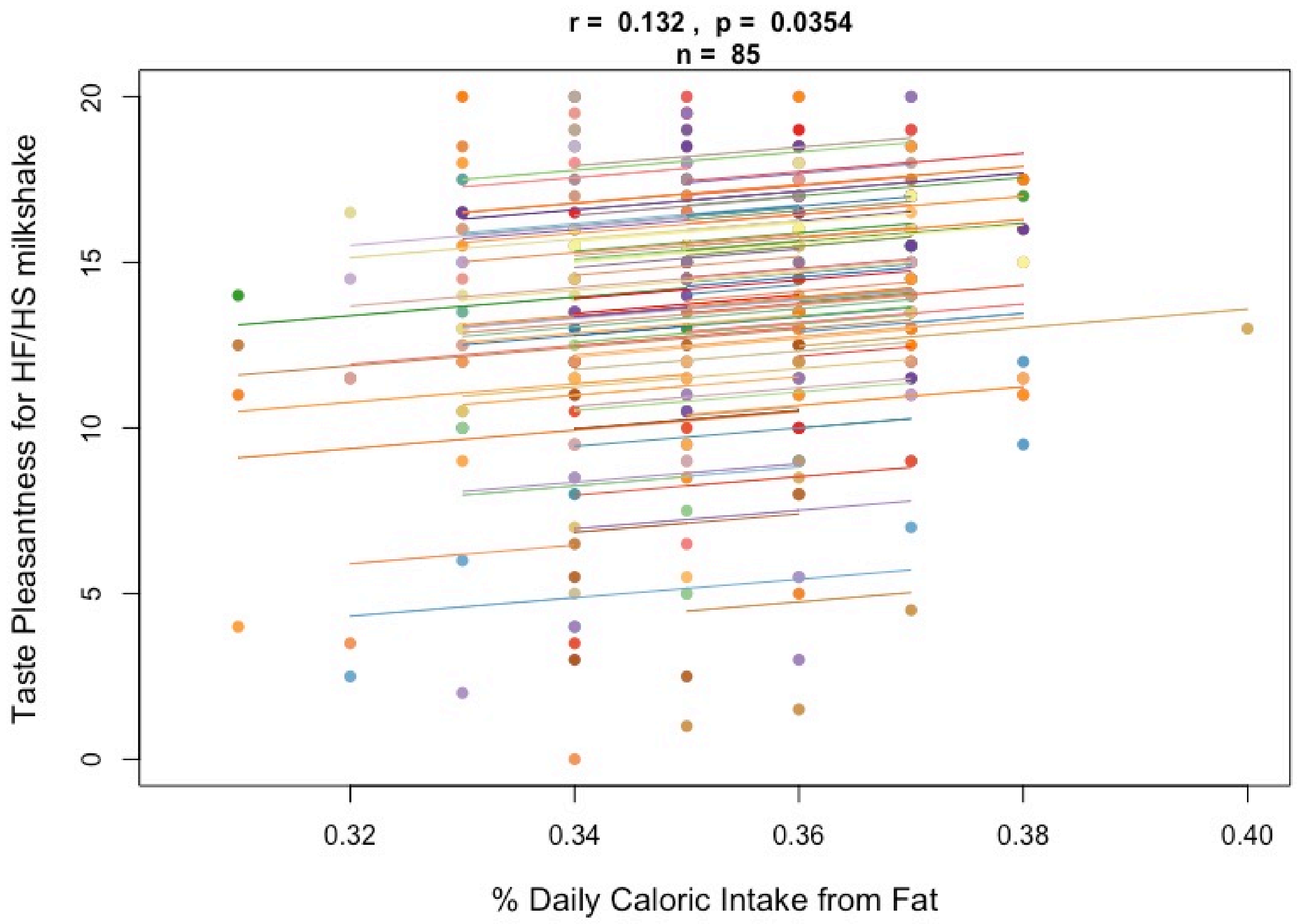

Supplementary Figure 2a. Repeated measures correlations between percent daily caloric intake from fat and taste liking (pleasantness) for the high-fat/high-sugar (HF/HS) milkshake
