# Supplementary material for: Longitudinal Associations Between Taste Sensitivity, Taste Liking, Dietary Intake and BMI in Adolescents
Source: Front Psychol. 2021 Feb 18;12:597704. doi: 10.3389/fpsyg.2021.597704 (PMC7935517; doi:10.3389/fpsyg.2021.597704)
Supplement: Supplementary file 3 [file Image_3.PDF]

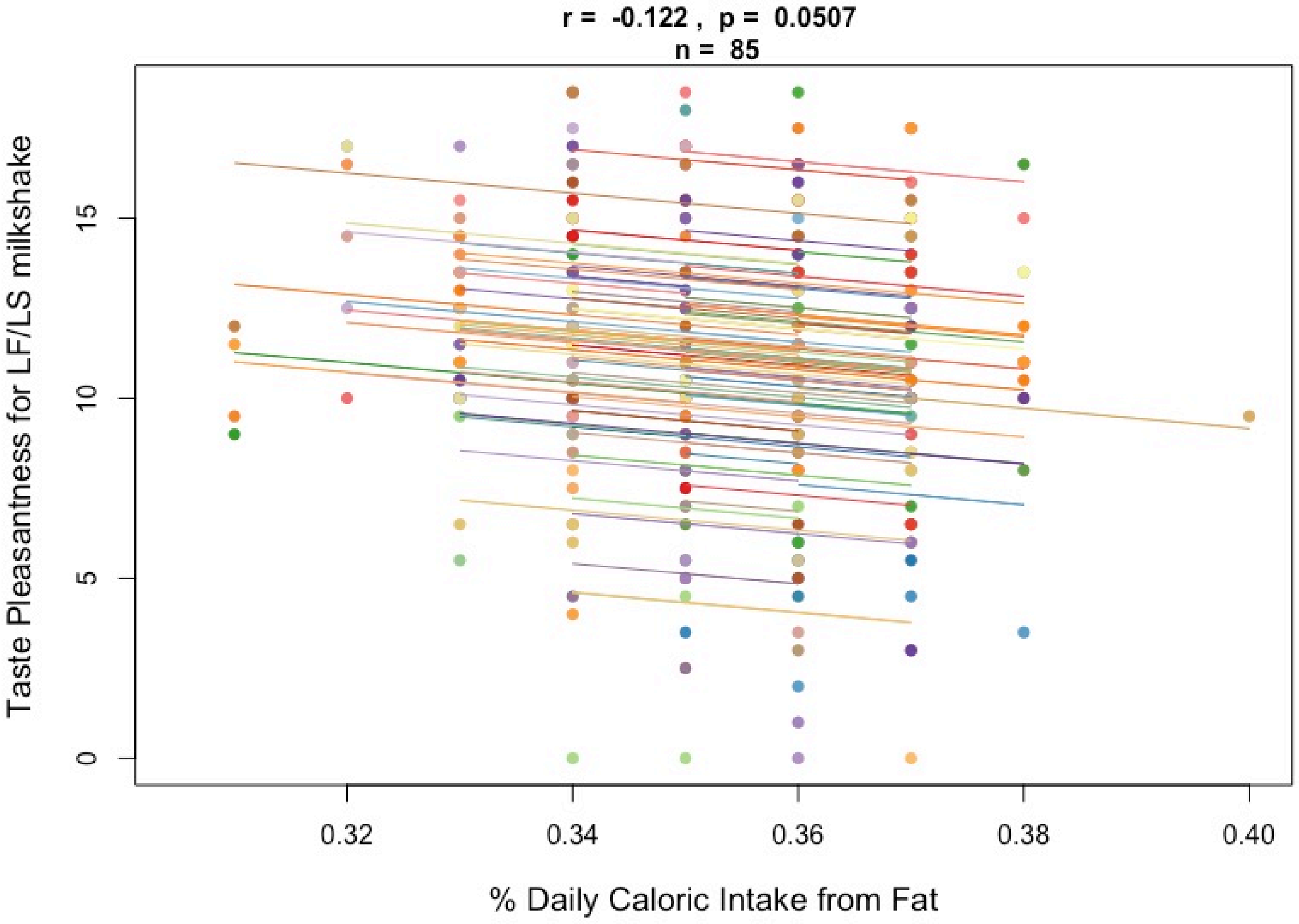

Supplementary Figure 2b. Repeated measures correlations between percent daily caloric intake from fat and taste liking (pleasantness) for the low-fat/low-sugar (LF/LS) milkshake
