# Supplementary material for: Longitudinal Associations Between Taste Sensitivity, Taste Liking, Dietary Intake and BMI in Adolescents
Source: Front Psychol. 2021 Feb 18;12:597704. doi: 10.3389/fpsyg.2021.597704 (PMC7935517; doi:10.3389/fpsyg.2021.597704)
Supplement: Supplementary file 4 [file Image_4.PDF]

Instructions And Scoring For The Triangle Taste Discrimination Tests

Instructions: Participants are presented with three different taste solutions at a time. Participants will be asked to indicate which taste solution is different from the rest. If they choose correctly, score this as a "1." If they choose incorrectly, score this as a "0."Record the participant's response using the form below. Start at level 3, and if the participant scores a "1" two times, proceed on to level 4 (down). If the participant scores a "0" twice, move backward (up) to level 2. Continue presenting the participant with the taste solutions until they have either failed to correctly identify the different taste solution twice and record the highest level correctly identified solution. For example: If a person fails to pick the different solution at level 3 two times, but correctly picks the different solution at level 2 (twice), they score a level 2.

Sugar

	Level 1: F, F, A	____	____	____
	Level 2: A, A, E	____	____	____
START HERE ->	**Level 3: E, E, B**	____	____	____
	Level 4: C, C, E	____	____	____
	Level 5: D, D, C	____	____	____

If wrong twice

If correct twice

Highest level correctly identified: Level_____

Fat

	Level 1: F, F, A	____	____	____
	Level 2: A, A, E	____	____	____
START HERE ->	**Level 3: E, E, B**	____	____	____
	Level 4: C, C, E	____	____	____
	Level 5: D, D, C	____	____	____

If wrong twice

If correct twice

Highest level correctly identified: Level_____
