# Supplementary material for: Longitudinal Associations Between Taste Sensitivity, Taste Liking, Dietary Intake and BMI in Adolescents
Source: Front Psychol. 2021 Feb 18;12:597704. doi: 10.3389/fpsyg.2021.597704 (PMC7935517; doi:10.3389/fpsyg.2021.597704)
Supplement: Supplementary file 5 [file Table_1.DOCX]

**Supplementary Table 1.** Subsample (n=85) Participant Characteristics and Behavioral Measures

|  | **Year 1 Visit (Baseline)** | **Year 2 Visit** | **Year 3 Visit** | **Year 4 Visit** |
| --- | --- | --- | --- | --- |
|  | *Count (Percent)* | | | |
| **Sex** |  | | | |
| *Male* | 39 (45.9) | | | |
| *Female* | 46 (54.1) | | | |
| **Race** |  | | | |
| *Asian* | 5 (5.9) | | | |
| *Black or African American* | 7 (8.2) | | | |
| *White* | 65 (76.5) | | | |
| *More than one race* | 4 (4.7) | | | |
| *Other or Missing* | 4 (4.7) | | | |
|  | *Mean ± SD (Range)* | | | |
| **Age (years)** | 15±1 (14-16) |  |  |  |
| **BMI (kg/m^2^)** | 21.1±2.4 (16.2-26.4) | 21.5±2.6 (16.8-28.3) | 21.9±2.8 (17.0-29.0) | 22.6±3.6 (16.2-40.6) |
| **BMI percentile*** | 55.7±24.8 (5.4-94.9) | 52.2±26.0 (4.6-94.7) | 50.3±26.6 (1.7-94.7) | 50.8±27.8 (0.5-99.7) |
| **Taste Sensitivity** |  | | | |
| *Fat* | 2.33±1.17 (0-5) | 2.33±1.18 (0-5) | 2.41±1.11 (0-5) | 2.69±1.30 (0-5) |
| *Sweet* | 2.86±1.00 (0-5) | 2.84±0.88 (1-5) | 2.94±1.12 (0-5) | 2.94±1.02 (1-5) |
| **Taste Liking** (pleasantness rating) |  | | | |
| *HF/HS* | 14.68±3.17 (7-20) | 13.35±4.22 (2.5-20) | 13.82±3.78 (1-20) | 12.84±4.49 (0-19.5) |
| *LF/HS* | 11.94±3.88 (1.5-20) | 11.92±3.96 (3-19.5) | 12.91±3.84 (0.5-19) | 12.69±3.52 (1-19.5) |
| *HF/LS* | 13.07±4.33 (1.5-20) | 13.19±4.40 (0.5-20) | 12.02±4.69 (1-19.5) | 12.24±3.99 (1.5-20) |
| *LF/LS* | 10.05±3.69 (0-17.5) | 11.39±3.90 (0-17.5) | 11.34±3.53 (2-18.5) | 11.32±3.56 (1-18.5) |
| **Hunger** |  | | | |
| Prior to Taste Sensitivity test | 8.52±4.92 (0-17.5) | 9.66±4.57 (0-17.5) | 10.41±4.06 (0-18) | 10.95±3.88 (0-17.5) |
| Prior to Taste Liking test | 11.09±3.91 (0-19.5) | 11.11±4.30 (0-19) | 11.40±4.17 (0-20) | 11.99±3.65 (1-18) |
| **Fullness** |  | | | |
| Prior to Taste Sensitivity test | 9.22±3.95 (0-19) | 8.18±3.77 (0-18.5) | 7.68±4.10 (0-18) | 7.59±3.83 (0-17.5) |
| Prior to Taste Liking test | 7.19±4.29 (0-20) | 6.84±4.42 (0-18.5) | 6.25±3.97 (0-14) | 6.36±3.86 (0-17) |

*n=82

HF/HS: high-fat/high-sugar milkshake; LF/HS: low-fat/high-sugar milkshake; HF/LS: high-fat/low-sugar milkshake; LF/LS: low-fat/low-sugar milkshake
