# Supplementary material for: Longitudinal Associations Between Taste Sensitivity, Taste Liking, Dietary Intake and BMI in Adolescents
Source: Front Psychol. 2021 Feb 18;12:597704. doi: 10.3389/fpsyg.2021.597704 (PMC7935517; doi:10.3389/fpsyg.2021.597704)
Supplement: Supplementary file 6 [file Table_2.DOCX]

**Supplementary Table 2.** Recipes For Fat And Sweet Taste Solutions In The Triangle Taste Discrimination Tests Used To Assess Taste Sensitivity

| **FAT Solutions** | | |
| --- | --- | --- |
|  |  | g/ml |
| **Solution A** (least amount of fat) | 1 cup low-fat (1%) milk + 2 tbsp Hershey’s chocolate syrup | 1.052 |
| **Solution B** | 1:1 ratio of solution A to solution C | 1.050 |
| **Solution C** | 1 cup whole (3.5%) milk + 2 tbsp Hershey’s chocolate syrup | 1.048 |
| **Solution D** | 1:1 ratio of solution C to solution E | 1.046 |
| **Solution E** | 1:1 ratio of solution C to solution F | 1.044 |
| **Solution F** (most amount of fat) | 1 cup half and half + 2 tbsp Hershey’s chocolate syrup | 1.041 |
| **SWEET Solutions** | | |
|  |  |  |
| **Solution A ^a^** (least amount of sugar) | 3 ¾ cups water, ¾ cup simple syrup **^b^** | 1.037 |
| **Solution B** | 1:1 ratio of solution A to solution C | 1.043 |
| **Solution C ^a^** | 3 ½ cups water, 1-cup simple syrup | 1.049 |
| **Solution D** | 1:1 ratio of solution C to solution E | 1.052 |
| **Solution E** | 1:1 ratio of solution C to solution F | 1.055 |
| **Solution F ^a^** (most amount of sugar) | 3 ¼ cups water; 1 ¼ cups simple syrup | 1.061 |

**^a^**: Solutions A, C, and F make 36 fluid ounces per recipe. For every 36 fluid ounces of solution created, ½ teaspoon Kool-Aid powder (fruit punch flavor) were added

**^b^**: simple syrup was prepared as 1:1 ratio of sugar to hot water

After preparation, 8 fl oz of each solution were used for the triangle taste test.
